# Defining animal welfare standards in hunting: body mass determines thresholds for incapacitation time and flight distance

**DOI:** 10.1038/s41598-018-32102-0

**Published:** 2018-09-13

**Authors:** Sigbjørn Stokke, Jon M. Arnemo, Scott Brainerd, Arne Söderberg, Morten Kraabøl, Bjørnar Ytrehus

**Affiliations:** 1 Norwegian Institute for Nature Research, P.O. Box 5685 Torgard, NO-7485 Trondheim, Norway; 2 Inland Norway University of Applied Sciences, Campus Evenstad, Postboks 400, 2418 Elverum, Norway; 3 Department of Wildlife, Fish, and Environmental Studies, Swedish University of Agricultural Sciences, SE-90183 Umeå, Sweden; 4 Alaska Department of Fish and Game, Division of Wildlife Conservation, 1300 College Road, Fairbanks, AK 99701 USA; 5 Department of Ecology and Natural Resource Management, Norwegian University of Life Sciences, P.O. Box 5003, NO-1432 Ås, Norway; 6 National Veterinary Institute, SVA, SE-75189 Uppsala, Sweden; 7 County Administrative Board, Box 22067, 104 22 Stockholm, Sweden; 8 Multiconsult Norway AS, Postboks 265 Skøyen, NO-0213 Oslo, Norway

## Abstract

Shooting is an important tool for managing terrestrial wildlife populations worldwide. To date, however, there has been few quantitative methods available enabling assessment of the animal welfare outcomes of rifle hunting. We apply a variety of factors to model flight distance (distance travelled by an animal after bullet impact) and incapacitation from the moment of bullet impact. These factors include body mass, allometric and isometric scaling, comparative physiology, wound ballistics and linear kinematics. This approach provides for the first time a method for quantifying and grading the quality of shooting processes by examining only body mass and flight distance. Our model is a universally applicable tool for measuring animal welfare outcomes of shooting regimes both within and among species. For management agencies the model should be a practical tool for monitoring and evaluating animal welfare outcomes regarding shooting of mammalian populations.

## Introduction

At present, in many ecosystems, wildlife managers depend upon hunters using rifles to kill wildlife to keep the number of animals at levels relatively adapted to food resources, space needs, and acceptable levels of impact on human infrastructure and production. Hunting might be professional, related to subsistence or recreational with intrinsic cultural value. In any case, there is a growing concern about the animal welfare outcomes of shooting, both among the hunters themselves, wildlife managers, animal welfare authorities and the public at large^1^. However, it is inherently difficult to objectively and quantitatively measure degrees of animal welfare in the shooting situation, and only few efforts have been made^1–4^.

Ideally, shooting of mammals should cause “the least animal welfare harms to the least number of animals”^5^. This should be quantified in terms of the shortest possible duration and the lowest possible intensity of suffering to the animal^6,7^. Intensity is subjective and difficult to evaluate, which leaves us with time to death (duration or time to insensibility) as a key parameter for assessing animal welfare outcomes^1,8^. Unfortunately, it is very difficult to gather data on elapsed time between the striking of the bullet and incapacitation in relation to body mass. This is because large mammals frequently disappear in dense cover before they collapse, which hinders accurate recording of timespans.

However, despite considerable interest and suggested “Best Practices” for hunting, there is a persistent lack of scientific studies that quantify animal welfare aspects (wounding rates) in hunted populations of terrestrial mammals^1^. This paucity of information is an impediment to developing tools to reduce suffering and wounding in one of the most common and important interactions between man and individuals of hunted wildlife species.

In this paper we describe a model based on body mass as a determinant of the time to incapacitation and the flight distance after an animal is struck by a bullet. This model enables us to define animal welfare outcomes for shooting and wounding of game mammals.

## Background

Each year millions of mammals are killed worldwide with firearms using expanding bullets. In compliance with “Best Practice” in most countries, the hunters target the thoracic area to induce fatal hemorrhaging from the highly vascularized lungs, the heart or the large thoracic blood vessels. These animals die from blood loss leading to hypovolemic shock, insensibility and inevitably death (for definition and classification of shock, see^9,10^). After an animal has been shot, locomotion may continue until incapacitation (see definition below) occurs due to exsanguination^11^. The time to incapacitation and subsequent death will depend on the rate of hemorrhage from disrupted vessels^12,13^. However, flight distance is a much easier and more practical parameter to deal with than duration and therefore it makes sense to search for a link between these two parameters. Most physiological properties of mammals (including blood perfusion rate) scale to body mass^14^. This relationship suggests there may be a relationship between body mass and flight distances in game mammals shot with expanding bullets.

In a preliminary report, Stokke *et al*.^15^ found that body mass was positively correlated with flight distances of shot moose (*Alces alces*), brown bear (*Ursus arctos*), lynx (*Lynx lynx*) and red fox (*Vulpes vulpes*). Gremse and Rieger^16^ and Kanstrup *et al*.^17^ observed similar trends in studies on red deer (*Cervus elaphus*), wild boar (*Sus scrofa*), mouflon (*Ovis aries musimon*), chamois (*Rupicapra rupicapra*), fallow deer (*Dama dama*), and roe deer (*Capreolus capreolus*). However, there are no published studies that examine the relationship between body mass and time to incapacitation and death due to hemorrhaging.

In this paper, we use body mass (moose, brown bear, roe deer and red fox), allometric scaling, wound ballistics, and comparative physiology to develop a mathematical model estimating time to incapacitation in relation to body mass for mammals shot with expanding rifle bullets. By applying linear kinematics and field hunting data we convert model output from time to incapacitation to flight distance in relation to body mass (calibrating the model). Furthermore, we show how the model can be applied in wildlife management to determine whether shooting of a given species meets or exceeds animal welfare standards. We also define a flight distance threshold for defining whether an animal is wounded or not.

Applied terms:

- Flight distance: the actual distance traveled by an animal from where it was initially struck by the first bullet to where it fell incapacitated^15^ (i.e. not the straight distance between these two locations, but the distance along the route the animal followed).
- Incapacitation: the state where a wounded animal is recumbent, immobile, and regarded as unconscious due to the inability of an observer to assess physiological responses from a distance. However, an incapacitated animal may not be clinically dead.
- Time to incapacitation: the time interval between the first shot being fired at an animal and the moment the animal is incapacitated^1^.
- The bleeding tissue volume (BTV): the permanent wound cavity + the extravasation zone where significant bleeding occurs (see also the wound ballistics section).

### Scaling of biological processes

Although terrestrial mammals vary greatly in body mass, from a 2 g Etruscan shrew (*Suncus etruscus*) up to a 6-metric ton African savanna elephant (*Loxodonta africana*), many of their fundamental biological and physiological processes can be described as allometric equations based on body mass. Allometric relationships are frequently presented quantitatively as a power function of the form (1):

1 $$y=a{M}^{b},$$

where *y* is some variable of structure or function, *M* is body mass and *a* (scaling factor) and *b* are constants derived empirically^14,18,19^. The allometric exponent *b* is the slope of the straight regression line when the equation is plotted with log-transformed values in the following rewritten manner (2):

2 $$log\,y=log\,a+b\cdot log\,M$$

Note that, in physical nomenclature, the power function is a scaling relation describing how *y* scales with M.

The classical power function is the allometric relationship between body mass and metabolic rate^20^. Even though the value of the allometric exponent b has been disputed^21–24^, it is widely accepted that a value of 0.75 (i.e. ¾ power) is valid for terrestrial mammals (see review^25,26^). This means that the rate of transformation of energy decreases with increasing body mass. Furthermore, rates of most biological and physiological processes can be described by a limited set of values of *b*, typically simple multiples of ¼^27^. If some variable of structure or function maintains the same fraction of body mass with increasing size, the allometric exponent *b* = 1 and the relationship is termed isometric scaling.

### Wound ballistics

Based on Maiden’s review^13^, ballistic research deals with four areas (quote): (1) Internal ballistics – the study of accelerating projectiles inside the barrel of a gun; (2) Intermediate ballistics – the behavior of projectiles when leaving the barrel muzzle; (3) Exterior ballistics – the study of interactions between projectiles and air before impacting the target; (4) Terminal ballistics – the study of projectiles penetrating a medium denser than air^28^. Wound ballistics is a sub-domain of terminal ballistics and is concerned with projectiles penetrating living tissue^13,28,29^.

Most bullet designs intended for hunting expand upon impact. Expansion is characterized by a mushroom-like frontal increase in the cross-sectional area of the bullet at the moment of impact.

The commonly used heterogenous bullets consist of a lead core covered with a copper jacket except for the leading lead tip. Heterogenous bullets expand due to the force acting on the exposed lead tip upon impact with tissue. The drag forces generated by the stagnation pressure at the tip of the exposed bullet exceed the yield limit for lead, which then behaves like an incompressible fluid^28^. Thus, pressure disperses within the floating lead and acts on the jacket from inside the bullet, causing it to burst^28,30^. Deformation is extremely rapid and occurs within 0.1 ms^28,30^. Homogenous bullets (solid copper or copper alloys) expand in a similar manner, due to the same mechanisms, as long as the hollow cavity at the tip of the bullet is large enough for viscous pressure to enter^28^.

The increased frontal area of the bullet crushes and lacerates the tissue it strikes during penetration. Simultaneously, tissue is impelled radially in relation to the velocity vector, as momentum is imparted from the projectile to the soft tissue, and it undergoes elastic deformation as it is stretched and compressed^31^. This creates a temporary empty space behind the bullet, termed the temporary wound cavity. The vacuum and the elastic energy imparted to the tissue rapidly forces the displaced tissue to recoil towards its initial position, thus generating a brief oscillation^28,32–36^. The residual wound channel, a cavity filled with blood, damaged tissue, and contaminants sucked in from the outside, is termed the permanent wound cavity^11,37,38^. The permanent wound cavity is surrounded by a transition zone, the extravasation zone, where crushing and laceration by direct contact with the bullet not has occurred, but where hemorrhages result from the distention of the temporal cavity, inflicting damage to blood vessels through overstretching and shearing effects (due to the heterogeneity of the involved tissues)^28,31^. The kinetic energy of the striking bullet is approximately proportional to the size of the temporary cavity and modulated by the elasticity of the penetrated tissue^28,31^. The maximum expansion of the temporary cavity reflects the potential energy stored in the tissue and equals the work done to create the deformation. MacPherson^39^ states that the potential for this energy to cause wounding depends on four factors:

1. The magnitude of the stored energy in the tissue
2. The ability of the tissue to sustain strain
3. The size of the organ structure
4. Anatomical constraints to tissue movement

The extent of permanent damage caused by a bullet depends on the elasticity of the tissues being penetrated. If the stored energy exceeds the elastic limit of the tissue, it will rupture and permanent wounding results. Elastic tissues, such as muscle, skin, blood vessels, and lungs, can absorb much of the energy deposited by a bullet and tend to recoil towards the wound channel^37,39,40^. In contrast, inelastic tissues, such as liver, kidney, and brain, tend to disrupt from penetrating projectiles^31,41,42^. If the organ (or body) is critically small, all tissues will be stretched beyond the elastic limit of the organ (body) causing it to rupture. For organs (bodies) larger than this critical size, tissue damage primarily occurs by crushing, tearing, and stress^39^. Thus, the primary factor causing permanent wound cavity in soft tissue like lungs will mainly be crushing, rather than radial stretching, as long as organ size exceeds the critical size^31^. This suggests that the radial area of hemorrhage in lungs will not vary much with organ size, if they are so large that the tensile forces do not exceed the elastic limit of the organ.

### Cause of death of mammals struck by hunting bullets

If an animal dies immediately after being shot with a hunting bullet, the only probable mechanism of death is direct trauma to the central nervous system (CNS). A bullet impacting major blood vessels, or the heart will cause fatal hemorrhage, but there is never an instant loss of consciousness^12^. In cases of CNS trauma where the bullet traumatizes vital parts of the brain, i.e. upper cervical spinal cord, the brain stem, the cerebellum, large parts of diencephalon and midbrain, the motor cortex and major part of motor conduction, and causes sufficient tissue trauma, the animal is instantly incapacitated due to loss of consciousness and will subsequently die from heart and respiratory arrest and hypoxia^43^. If the bullet strikes high in the neck, traumatizing the spinal cord cranially to or at C4-C7, the origin of the phrenic nerves in domestic species^44^, the animal will be knocked down but will remain conscious. Because the phrenic nerves are the sole motor innervation of the diaphragm^45^, it will be paralyzed. The animal will die from respiratory failure and subsequent brain hypoxia, but this may take several minutes. Other impacts in the spinal cord, i.e. caudally to C7, are not fatal, unless large blood vessels are also traumatized. The animal may, however, be incapacitated and may, depending on the point of impact, remain recumbent or in a “dog-sitting” posture, due to paralysis of motor nerves. Also, the animal may remain fully conscious and aware of its surroundings and sensitive to pain arising from body parts with intact sensory neurons cranially to the point of spinal cord trauma.

Exsanguination is the major cause of death in many hunted animals, because hunters, in accordance to codes of practice, routinely target the central thoracic area. An expanding bullet will usually penetrate the thoracic cavity, causing trauma to the heart, lungs, and/or major blood vessels with subsequent fatal hemorrhaging. Suboptimal impacts in other body parts, such as the neck or abdomen, may also cause fatal hemorrhage if a large vessel is severely lacerated or a well-perfused organ, such as the liver, or a kidney is ruptured. Death due to blood loss is in practical life never instantaneous and the time from bullet impact to incapacitation principally depends of the rate of hemorrhage. The mechanism of death is circulatory collapse, more accurately hypovolemic shock caused by hemorrhage^9,10^, with subsequent brain death due to tissue hypoxia. Empirical data (mean values) in moose indicates that it takes 10 seconds after a bullet penetrates the heart and 30 seconds after penetration of both lungs before an animal is incapacitated^46^. It is important to notice that our model solely address penetration of the thoracic area.

### Hypothesis

We considered that three principal parameters determine the time to incapacitation when an animal positioned at the horizontal plane is dispatched with an expanding bullet penetrating both lungs centrally and perpendicularly to its longitudinal axis;

1. total blood volume;
2. blood circulation time and;
3. wound size in relation to body width (i.e. body mass).

Furthermore, we presumed that the diameter of the BTV remains quite unchanged in lung tissue as long as the lung mass is large enough to avoid total rupture due to the temporary cavity (see wound ballistics section). For practical purposes, we considered this volume to have a cylindrical shape. Additionally, we recognized that total organ blood volumes can be combined with organ perfusion rates to calculate blood circulation times^27,47^.

Consequently, while the BTV diameter remains virtually constant regardless of lung size, effective wound track cylinder length (i.e. body width) scales allometrically to body mass^48^. This suggests that the relative volume of the BTV to total body volume should diminish with body mass. Because blood volume remains unchanged with body mass^49^, blood circulation time increases with body mass^50^, and size of the BTV decreases with body mass, we hypothesize that bleeding rate will decrease with body mass. Thus, implying that time to incapacitation will increase with body mass according to some power function of form (3):

3 $${\rm{y}}={{\rm{aM}}}^{{\rm{b}}},$$

## Development of the Model and Estimation of Parameters

### Modeling synopsis

First, we developed a theoretical mathematical model based on reported allometric relationships available from the literature. Our power function model describes time from bullet penetration until a critical blood volume has been lost and incapacitation occurs, in relation to body mass. To create the model, we needed to incorporate two unknown variables: (1) the amount of blood flowing out of the injured vessels of the BTV, and; (2) the radius of this cylinder. Critical levels for loss of blood causing incapacitation or unconsciousness are described in medical literature^13,51,52^, but the radius of the BTV is unknown. To estimate the dimension of this hypothetical cylinder, we applied empirical data from four mammalian species harvested in Fennoscandia (Finland, Sweden, and Norway) covering a body mass range from 4 to 600 kg. We used these data to build an empirical allometric power function model where flight distance scales with body mass. Because speed and distance covered are mutually dependent, we can transform this function into a power function connecting time until incapacitation to body mass. This approach gave us one theoretical and one empirical equation expressing how time to incapacitation scales with body mass. By using both equations, we could estimate the radius of the BTV and time to incapacitation in relation to body mass.

We used Visual FoxPro 9.0 SP2 to handle the data and for programming to execute all calculations. All graphs were made using SigmaPlot 13.0.

### The basic theoretical model

We assumed that the BTV more or less resembles a cylinder (see above), allometrically scaled to body width.

We applied three allometric relationships to build a mathematical model, describing blood flow through a hypothetical restricted BTV that represents partial blood loss from the total blood volume. Body mass is denoted as *M* (kg) in these equations. Blood circulation time, *t*, measured in seconds, scales to body mass in the following manner (4)^50^:

4 $$t=21{M}^{0.21}.$$

Respectively for blood volume, *V*, measured in ml (5)^49^:

5 $$V=55{M}^{0.99}.$$

Finally, body width, *W*, measured in cm, scales as follows (6)^48^:

6 $$W=3.55{M}^{0.47}.$$

We defined the BTV, *B*_*V*_, measured in ml, to be (7):

7 $${B}_{v}=\pi {r}^{2}\cdot 3.55{M}^{0.47},$$

Where *π* ≈ 3.14 and *r* is the radius of the cylinder. Then we can estimate the amount of blood, *P*, (0 < *P* < 1), flowing through the BTV during the time span *t* (8).

8 $$P=\frac{\pi {r}^{2}\cdot 3.55{M}^{0.47}}{21{M}^{0.21}\cdot 55{M}^{0.99}}t$$

Thus, we can estimate the time it takes until a critical amount of blood, *P*_c_, has streamed through the BTV, from which the blood is drained into the pleural cavities, adjacent lung tissue, the airways and the permanent wound tract (9).

9 $$t={P}_{c}\frac{1155}{3.55\pi {r}^{2}}{M}^{0.73}=\frac{{P}_{c}}{{r}^{2}}103.56{M}^{0.73}$$

This expression (9) therefore estimates the time it takes to incapacitate an animal in relation to body mass, if the value of *P*_*c*_ indicates critical blood loss (i.e. cerebral hypoxia) and *r* denotes the radius of the cylindrical BTV. Critical values for *P*_*c*_ has been suggested in the literature^13,51,52^, whereas *r* is totally unknown but can be estimated empirically, as shown below.

### Development of the empirical model

To solve the problem of the unknown *r*, we needed another equation of analogous form, expressing time to incapacitation in relation to body mass. Over many years, we have collected data from hunters and harvested game in Fennoscandia in order to provide wildlife managers with information regarding hunting practices^15,53^. Because the flight distance parameter conveys information about elapsed time, we could use this information to work out an indirect solution for the unknown *r*.

#### Data selected to develop the empirical model and related assumptions

Data were collected from questionnaires we distributed to hunters in Fennoscandia: Moose (Finland, Sweden, and Norway 2004–2006) n = 5245; brown bears (Sweden 2006–2010) n = 637; roe deer (Norway 2014–2015) n = 38; red fox (Norway 1993–2009) n = 160. For all studies, each hunter completed one form per harvested animal. A broad range of information is available in the datasets (such as caliber, bullet type and weight, cartridge and so on), but for the present study, the following information is relevant: flight distance (m), number of impacting bullets, the angle of the bullet trajectory in relation to the animal’s longitudinal axis, penetrated organs and bones, and whole or slaughter mass. Flight distance was only recorded for retrieved animal carcasses. Measurements were obtained by pacing out or by using a GPS. Hunters were asked to locate the spot where the animal was struck by the bullet and from that point start pacing out along the track of the animal until they arrived at the incapacitated animal. This route, covered by the shot animal, was recorded as flight distance in the form. We did not ask for wounding data per se, as our main interest was to record successful shooting data.

**Figure 1:**
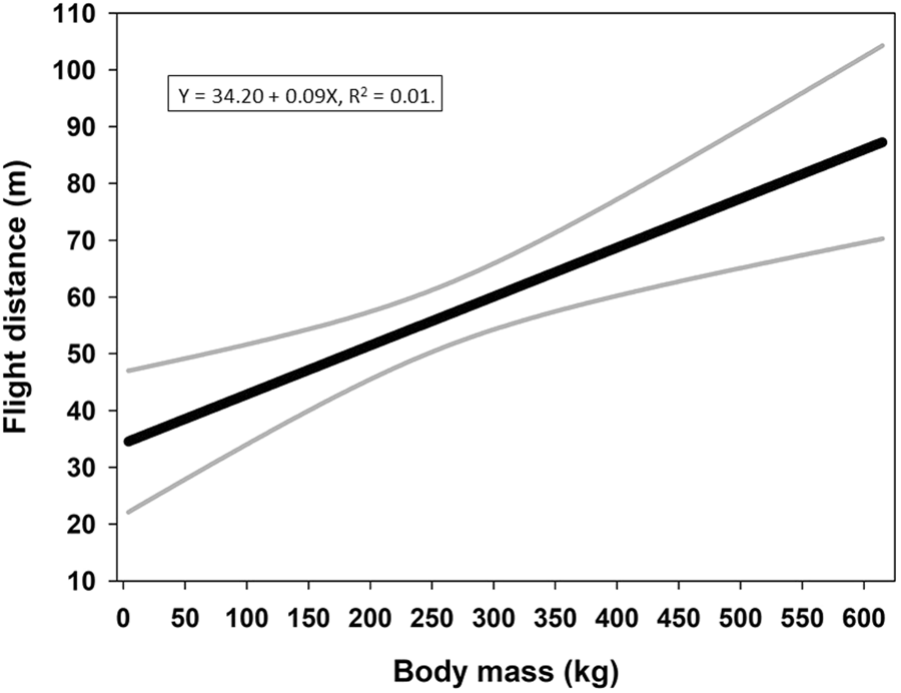
Regression of flight distances in relation to body mass with 95% confidence intervals. All available data are included in the regression. This graph represents a general trend between body mass and flight distance, irrespective of penetrated organs or number of impacted bullets.

We have data from all age classes for all the studied species, assuring a continuum of body masses. A linear regression of all available data suggested that flight distance increases with body mass (Fig. 1). For this study, we selected body masses and flight distances to generate the power function that scales flight distance with body mass. For body masses of foxes, we used whole body weights. The same applies for body masses of most bears. For all roe deer, and moose and some bears, we converted slaughter weights, *W*_*s*_, to live weights, *W*_*l*_. For roe deer and moose, the following relationship was applied^54^ (10):

10 $${W}_{l}=\frac{100\cdot {W}_{s}}{52}$$

For bears, we used the following relationship when converting to live weights (11)^55^:

11 $${W}_{l}=4.63+1.49\cdot {W}_{s}.$$

Because our theoretical model (9) was based on bleeding rates, we included only records fulfilling the following requirements: (1) the target animal was dispatched with one bullet; (2) the bullet trajectory described an angle of incidence ≤ 45° (in relation to the longitudinal axis of the animal in the horizontal plane), and; (3) both lungs were penetrated. We selected only cases where both lungs were penetrated by bullets because blood flow to the lung remains virtually unchanged in relation to body mass (12):

12 $${\rm{Y}}=0.107\cdot {{\rm{M}}}^{0.9}$$

This makes this organ suitable for comparison of bleeding rates among mammal species covering a wide range of body masses^27^. This strict selection caused a notable reduction in usable records for all mammals (moose-901, brown bear-59, roe deer-23, fox-74: see supplementary dataset).

The empirical model expresses time to incapacitation based on a conversion of flight distances. Because a recorded flight distance equal to zero does not convey any information about time and rate of exsanguination, we excluded these records, except for mammals with body masses ≤30 kg (see the wound ballistics section).

Flight distances are based on data from animals dispatched with cartridge types representing 22 different calibers (52 cartridges are represented in the final dataset: see supplementary information on applied cartridges). To simplify modeling, we assumed that different cartridge or calibers types did not affect flight distances. Likewise, we assumed that different bullet constructions did not affect flight distance.

#### Estimation of r

We log(10) transformed flight distance and body mass and regressed the data to obtain the following relation (13):

13 $$y=a{M}^{b},$$

where *y* is the flight distance, *a* is a constant, *M* is body mass (kg), and *b* is the allometric exponent. Traveling speed over the recorded flight distances probably varies according to some modulating factor related to body mass. This relationship might follow a simple rule such as the following (14):

14 $$v(M)=\frac{a{M}_{m}^{b}}{t}{(\frac{M}{{M}_{m}})}^{k},$$

where *v*(*M*) is speed for a given body mass *M*, *M*_*m*_ is the reference body mass with traveling time *t*, and *k* is a modulating factor. However, to keep the modeling as simple as possible, we assumed that all individuals, regardless of species and body mass, moved with the same speed (i.e. *k* = 1 in equation 14).

Because we know the approximate time to incapacitation for adult moose^46^, we can estimate time to incapacitation, $\hat{t}$, in seconds for any body mass by converting values according to the following relationship (15):

15 $$\hat{t}=\frac{T\cdot a{M}_{i}^{b}}{a{M}_{m}^{b}},$$

where *T* is time (s) to incapacitation for adult moose with body mass *M*_*m*_ (kg) and *M*_*i*_ is the body mass (kg) for which time (s) to incapacitation is calculated. Thus, corresponding body mass and time to incapacitation could be estimated for each recorded body mass.

Finally, the transformed set of interdependent times and body masses could be log(10) transformed and regressed to obtain the second expression for time, $\check{t}$, to incapacitation in relation to body mass (16):

16 $$\breve{t}={a}_{e}{M}^{{b}_{e}},$$

where *a*_*e*_ is a constant and *b*_*e*_ is the allometric exponent, as in the theoretical model (9), and the subscript letter *e* indicates that the values are based on empirical data from four mammalian species.

If the value of *b*_*e*_ is sufficiently close to 0.73 (the allometric exponent in the theoretical model (9)), we can argue that the following relationship is applicable (17):

17 $$103.56\cdot \frac{P}{{r}^{2}}={a}_{e}\,\to \,r=\sqrt{\frac{P\cdot 103.56}{{a}_{e}}}$$

Thus, by using equation 17 we could estimate how *r* varies in relation to *P*.

We applied bootstrapping to address the uncertainty in the estimation of the parameters in the modeling process. From the original dataset of corresponding flight distances and body mass, we generated 1000 bootstrap samples (bootstrap 1) with replacement. For each bootstrap sample, we regressed the sample according to equation 13. Then we recalculated all flight distances in the sample to time to incapacitation using equation 15. In doing so, we depended on Röken’s^46^ observed value for *T* (30 s) and *M*_*m*_ (body mass of adult moose). However, due to uncertainty of these values, each bootstrap sample (from bootstrap 1) was recalculated 5 times with variable *T* values increasing from 20 to 40 seconds in steps of 5 seconds. For each recalculation of the bootstrap sample (from bootstrap 1), an average value for *M*_*m*_ was determined by bootstrapping (bootstrap 2) among adult moose body masses from our original dataset. The number of bootstrap samples in bootstrap 2 equaled the number of adult moose (77) used in the Swedish study^46^. These final bootstrap samples where then regressed according to equation 16 to obtain bootstrap values of *a*_*e*_ and *b*_*e*_ respectively. To generate a bootstrap distribution for *b*_*e*_ we repeated the described procedure 1000 times.

**Figure 2:**
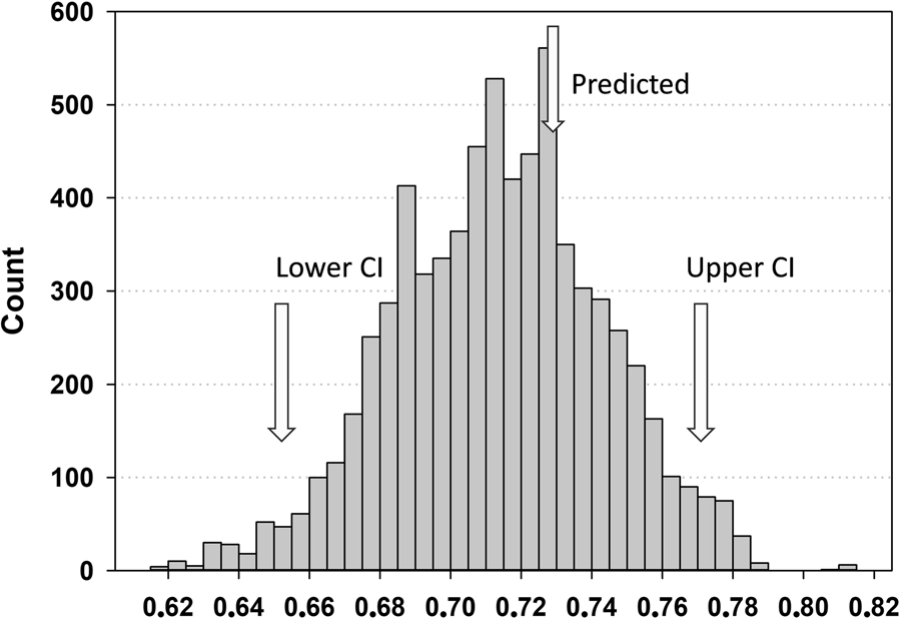
The distribution of bootstrapped allometric exponents generated by sampling with replacement. Predicted, upper and lower confidence intervals (95%) for the bootstrap distribution are indicated with arrows.

**Figure 3:**
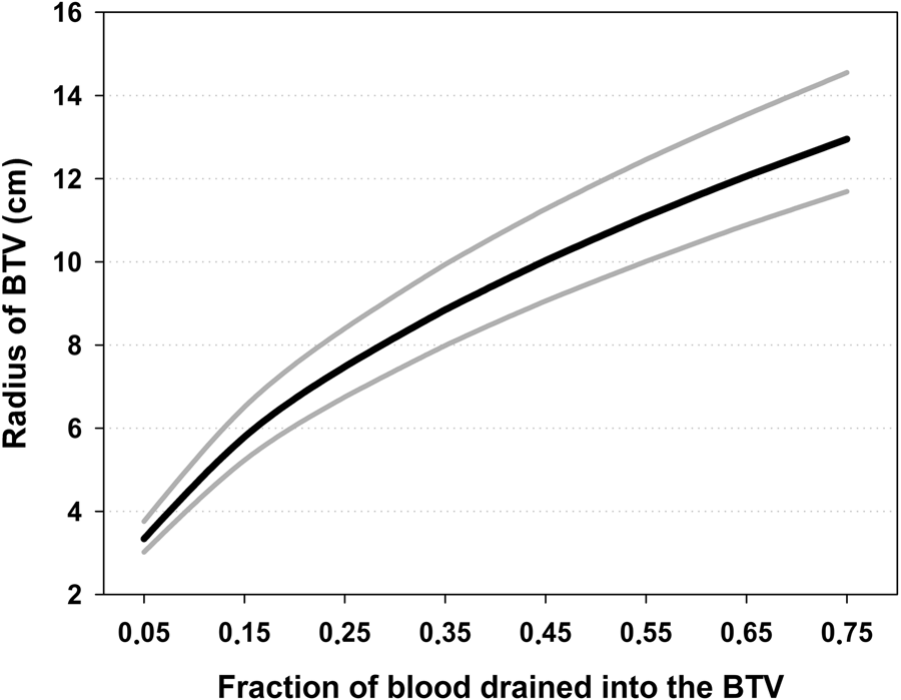
Simulation of the fraction of total blood volume percolating from the circulation into the BTV in relation to the radius of the bleeding cylinder. The black central line represents the median and the boundaries for the interquartile range are exhibited with gray lines.

The allometric exponent (0.73) from our theoretical model (9) was included in the 95% confidence interval for the bootstrap distribution of *b*_*e*_ values (Fig. 2). Thus, we could apply equation 17 to calculate values for *r* using all *a*_*e*_ values with 8 *P* values per *a*_*e*_ value, covering the interval from *P* = 0.05 to 0.75 using step values of 0.1. The median and 25^th^ and 75^th^ percentiles were calculated for each discrete value of *P* and displayed with *r* values for the BTV in Fig. 3.

**Figure 4:**
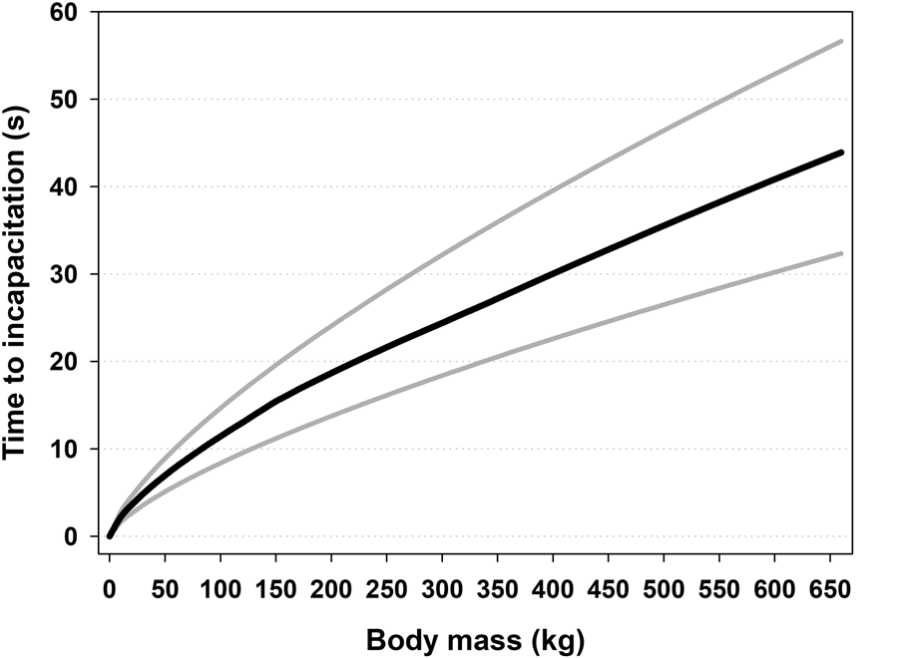
Simulated times to incapacitation in relation to body mass. The black central line represents the median and the boundaries for the interquartile range are exhibited with gray lines.

Finally, we applied the theoretical model (9) by using the interdependent values for *P* and *r*, generated from the bootstrap procedure described above, to estimate time to incapacitation for every 10 kg from body mass 0 kg (included to tag the graph to the axes) up to and including 650 kg. We applied *P*-values from 0.15 to 0.35, because this covers an interval (0.2–0.3) of blood loss supposed to incapacitate an animal^13,51^. Therefore, we assumed that the median values per weight class estimated from the generated values for time to incapacitation would be the best approximation to the real values. The median values are shown in Fig. 4 along with the 25^th^ and 75^th^ percentiles.

**Figure 5:**
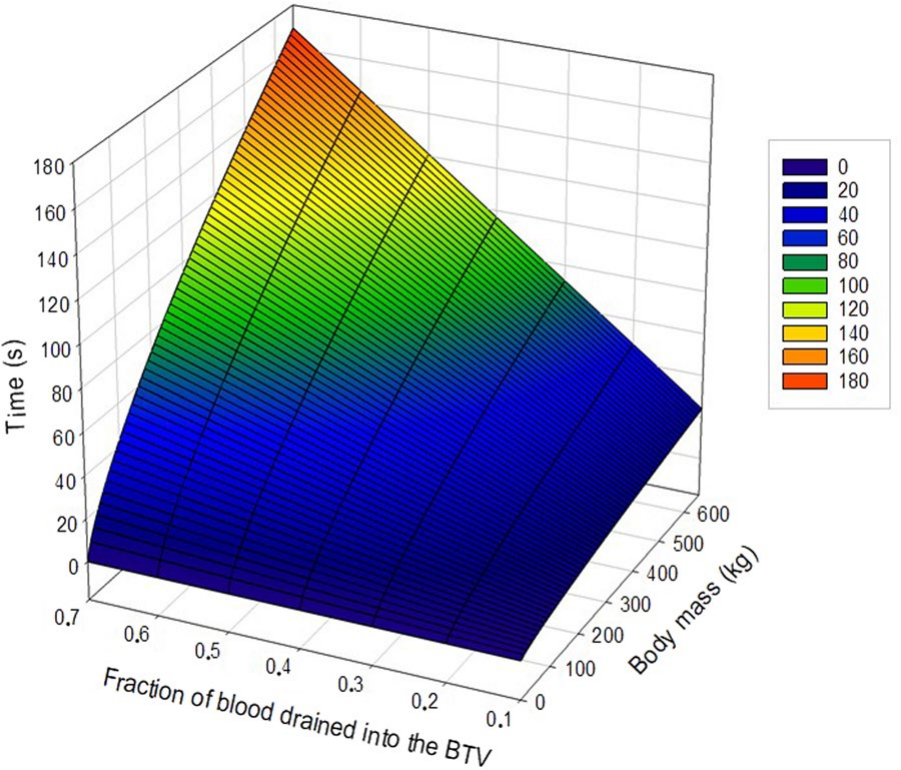
The relationship between body mass and the time it takes before a given amount of total blood volume being lost from circulation into the BTV. The relationship is simulated for a BTV with a radius of 7 cm. To the right of the graph are discrete color-codes for time intervals.

To illustrate the relationship between body mass and the time it takes before a given amount of total blood volume is lost from the BTV, we evaluated the theoretical model (9) by keeping the radius of the BTV constant (*r* = 7 cm.). Body mass varied between 0 and 650 kg and the fraction of blood drained into the BTV varied from 0.1 up to and including 0.7 (Fig. 5).

## Result Summary

Our modeling approach was based on the observed trend of diminishing flight distances with decreasing body mass for moose, brown bear, roe deer, and red fox harvested in Fennoscandia (Fig. 1). This result suggests that time to incapacitation should increase with body mass. We developed a theoretical allometric mathematical power model (9) descibing time to incapacitation in relation to body mass, blood loss, and radius of the BTV for game dispatched with firearms propeling expanding bullets.

$$t=\frac{{P}_{c}}{{r}^{2}}103.56{M}^{0.73}$$

The theoretical equation (9) suggests that there is no linearity between time to incapacitation and body mass. Rather, time to incapacitation scales positively with body mass in an allometric manner. Thus, the theoretical model (9) describes the time needed for a given amount of blood to perfuse the BTV in relation to body mass and radius of the BTV. However, we are not aware of any measurement for the BTV (i.e. *r*). Hence, we developed an alternative allometric power model (16) for time to incapacitation, using an indirect method, based on our field data from hunting of the four game species.

$$\breve{t}={a}_{e}{M}^{{b}_{e}}$$

The distribution of bootstrapped allometric exponents, *b*_*e*_, from the empirical model (16) is shown in Fig. 2. The value of our predicted theoretical allometric exponent (0.73) is situated close to the middle of the 95% confidence interval for the distribution of the empirical allometric exponents (Fig. 2). Thus, we could simulate the fraction of blood, *P*, drained into the BTV in relation to the radius, *r* of the BTV (Fig. 3).

$$r=\sqrt{\frac{P\cdot 103.56}{{a}_{e}}}$$

The model (17) intuitively predicts that an increased percolation of blood into the BTV demands an enlarging of the radius in the bleeding cylinder (Fig. 3). Hovewer, this relationship is not linear and the median value for the radius increases more rapidly for small amounts of blood loss per time unit than for larger ones (Fig. 3).

The theoretical model (9) could then be used to simulate time to incapacitation in relation to body mass with bootstrapped parameters (Fig. 4). The gray lines represent the 25^th^ and the 75^th^ percentiles and represent the uncertainity of parameter estimation. The theoretical model (9) suggest that time to incapacitation increases with body mass but with a decelerating trend (Fig. 4).

## Relevance of the Model and Discussion

### Is the calibrated model concordant with real hunting observations?

Flight distance is regarded to be a key indicator of the killing effect of hunting bullets^15,56,57^. We are only aware of two papers describing flight distances of shot ungulates of differing body mass^16,17^. Both papers report an increasing flight distance with body mass, but they offer no explanation for this observed trend. Our model expresses time (s) to incapacitation in relation to body mass and implies body mass modulated dependency as we hypothesized. However, time is not a pragmatic unit for practical purposes and is not immediately compatible with the variable flight distance used by the before-mentioned authors. So, we needed to convert model output to represent flight distances (m) in relation to body mass. We applied the simple relationship (18):

18 $$f=vt$$

to convert the data, where *f* is flight distance (m), *v* is speed (m/s), and *t* is time (s), as expressed by our theoretical model (9). To calibrate the model, we applied our own and Röken’s^46^ data. According to Röken^46^, adult moose can sustain movement for 30 seconds after one fatal shot with a maximum flight distance of 300 m. Our own records indicate that adult moose cover an average distance of ~65 meters after a fatal shot. Thus, we assumed a “normal” speed of 2.2 m/s and a “maximum” speed of 10 m/s after fatal shots for adult moose. By multiplying our theoretical model (9) with these factors, we could estimate expected wounding thresholds (see next section) and flight distances for all body masses ≤ 650 kg.

The magnitudes of the parameters *r* and *P*_*c*_ are not well known. This applies in particular to *r*, which hitherto has never been estimated for live animals. Trinogga *et al*.^58^ examined 34 carcasses of shot and frozen ungulates (wild boar, roe deer, chamois, and red deer), using X-ray computed tomography, to measure permanent wound cavities (wound channels) in lung tissue. They found an increasing wound diameter with penetration depth, from around a 1 cm estimated marginal mean at impact up to approximately 7 cm at 10 cm depth. However, animals struck through the lungs experience pneumothorax, i.e. entry of air into the pleural cavity which eliminates the negative pressure that keeps the lungs expanded, causing the lungs to collapse. In addition, pressure from the intestines on the diaphragm will further compress the lungs after death. Thus, we expect a significantly larger effective wound channel in fully expanded lungs of live animals.

**Figure 6:**
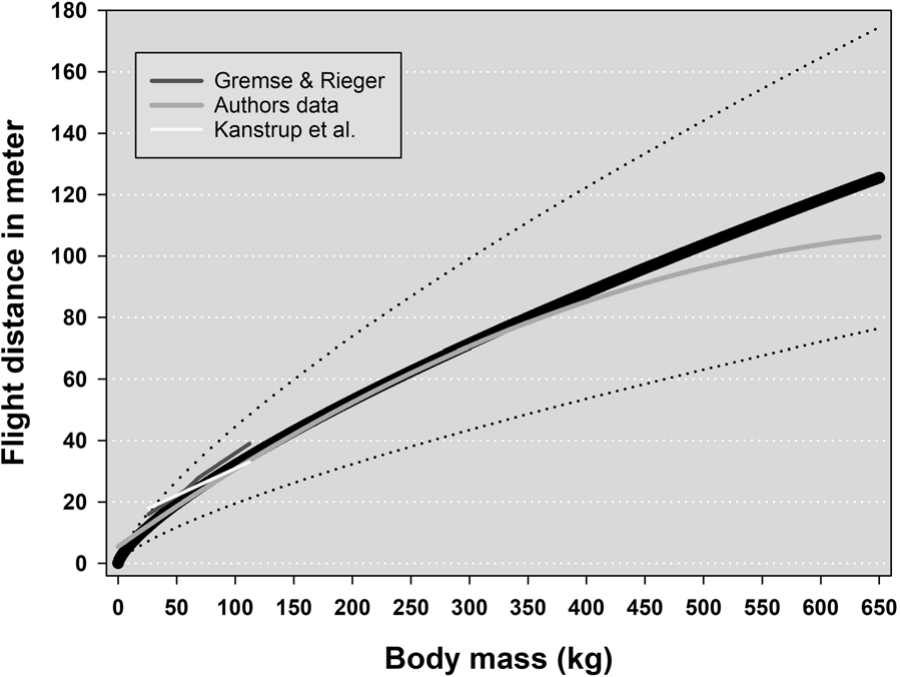
Expected flight distances suggested by the model for mammals with body masses ≤650 kg. The solid black line represents the average values and the stippled lines the standard deviation. The short white line exhibits average values of flight distances for four species (roe deer, fallow deer, wild boar, and red deer) with increasing body masses^16^. The short dark gray line shows average flight distances for roe- and red deer^17^. The long light gray line is the regression line representing flight distances for red fox, roe deer, brown bear, and moose calves, yearlings and adults (our own data).

Our modeling approach allowed simulating the relationship between *r* and *P*_*c*_ for fully expanded lungs (Fig. 3). In addition, Maiden^13^ suggested that an animal is rendered unconscious if blood loss is >20%, whereas McGuill and Rowan^51^ concluded that blood loss >30% causes hemorrhagic shock. Thus, we assumed that evaluating the model for values of *P*_*c*_ between 0.15 and 0.35 would include realistic values for *P*_*c*_. According to our modeling approach, these values suggest that *r* should vary between approximately 6–9 cm. (Fig. 3). We therefore used these interval values as input into our model to simulate corresponding flight distances. The model simulated flight distances in the following manner. First *P*_*c*_ values were increased stepwise with a step rate of 0.05. For each *P*_*c*_ value, flight distances were simulated for values of *r* covering the range of 6–9 cm. with an increasing step rate of 0.05. This operation was executed 651 times to cover body weights ≤ 650 kg. We assumed that the best approximation of real conditions would be the average flight distance per body weight. To illustrate the uncertainty of parameters, we calculated standard deviations per body mass (Fig. 6). Calibrated for expected flight distances (efd) the model (18) can be expressed as (19):

19 $${\rm{efd}}=1.14{{\rm{M}}}^{0.73}{\rm{.}}$$

To examine the relevance of our model (19), we compared model output with average flight distances reported by Gremse and Rieger^16^ in relation to average body mass (Fig. 6). We incorporated only reported flight distances resulting from high or low penetrations of lungs or heart. Kanstrup^17^ did not report body mass, only average flight distances. Therefore, we assumed normal continental body masses for roe and red deer when comparing with model output (Fig. 6). In addition, we plotted the regression line representing flight distances from red fox, roe deer, brown bear, and moose calves, yearlings, and adults (our own data). The calibrated model did indeed seem to predict expected flight distances remarkably well for all body masses (Fig. 6).

Our modeling approach suggested that the BTV was much larger than the radial dimensions of wound cavities measured by Trinogga *et al*.^58^ on frozen lungs. In fact, our model suggested that significant bleeding occurs within a volume twice the size (r ~ 7 cm) of the macroscopically visible wound cavity reported by Trinogga *et al*.^58^. This supports our presumption that the extravasation zone is a functional part of the wound. Furthermore, it indicates that the permanent wound cavities in fully expanded lungs are significantly larger before pneumothorax becomes evident. According to our calculations, it appears that the BTV must reach the estimated relative dimensions to cause a bleeding so profuse that circulatory collapse occurs within concordant time spans.

### Practical application of the model – defining thresholds for animal welfare outcomes

It is essential to accurately define the concept of wounding in order to assess the quality and animal welfare outcomes of a given shooting effort. Furthermore, such a definition should facilitate a quantitative measurement of the degree of wounding, so that different hunting methods, procedures, firearms, and ammunitions can be compared. Our model can easily be applied to establish a normative guidance for acceptable animal welfare outcomes for hunting systems across a wide range of body masses and game mammal species.

The lungs are a preferred area to target, because they represent the largest vital zone that leads to rapid incapacitation and death, due to extensive hemorrhaging^13^. The heart is also a preferred vital zone, but represents a smaller target. Penetration of the cervical column will cause instant incapacitation, due to complete paralysis, but if hemorrhaging is not profuse, the animal will remain able to sense and perceive pain and could live for several minutes before finally expiring. Thus, from an animal welfare point of view, penetration of both lungs induces reliable death. Therefore, we argue that our model represents a normative criterion for acceptable flight distances, if hunting is to be conducted according to animal welfare standards (Fig. 6).

**Figure 7:**
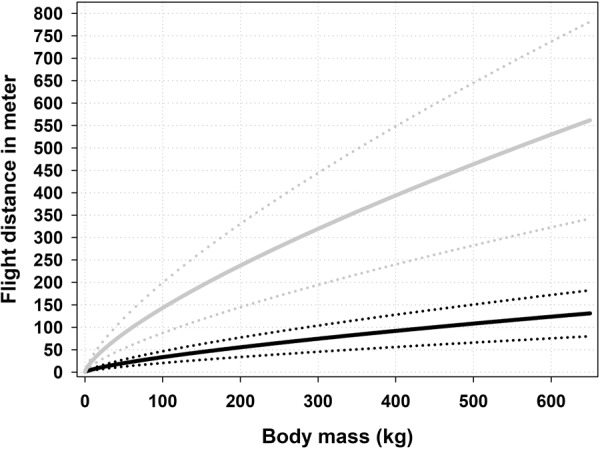
Wounding threshold (gray line) compared to expected flight distances (black line) suggested by the model for mammals up to body masses ≤650 kg. Stippled lines (standard deviation) represent uncertainty of parameter values.

To estimate threshold values for flight distances indicating wounding, simulations were made in the same manner as above, except we used a speed value of 10 m/s, instead of 2.2 m/s. This calibrated the model (18) to simulate extreme maximal flight distances, in relation to body mass, that can be covered by mammals after fatal shots (Fig. 7). Calibrated for maximal flight distances (mfd), our model (18) can be expressed as (20):

20 $${\rm{mfd}}=4.92{{\rm{M}}}^{0.73}{\rm{.}}$$

We argue that longer flight distances than these probably are due to penetration of either the marginal lung/heart area or other less sensitive organs and thus can be defined as wounding.

We suggest that our model can be used as a management tool, for instance to inform regulations for minimum caliber requirements for species and/or guidance to hunters where regulations are not restrictive. Hunters can quickly determine if they have made a clean shot simply by using Fig. 7 and looking up the predicted flight distance value corresponding to acceptable animal welfare or the corresponding threshold value for wounding in relation to a given animal body mass. If the animal has traveled further than the indicated wounding threshold, the hunter knows the animal is wounded. However, the hunter must rely on experience to judge how the suggested threshold distance might have been affected by such factors as terrain steepness, density of vegetation, animal stress level, and snow depth or other impediments. A thorough examination of the site where the animal stood when struck with the first bullet is still important.

Few attempts have been done to quantify animal welfare outcomes of mammal shooting. Hampton *et al*.^1^ designed a method based on data from shooting of European rabbit (*Oryctolagus cuniculus*). The method depends on both ante and post mortem information to enable a quantification of animal welfare outcomes. For instance, the method recommends using apparent time to death and pathological examination. Compared to our model, the approach of Hampton *et al*.^1^ is more time consuming, species specific, and depends primarily on independent observers. Our method demands only a rough estimate of the body mass of the pursued mammal and a measurement of flight distance. Thus, it can easily be applied in the field and interspecific comparisons can be made without extended species-specific information. However, contrary to Hampton *et al*.^1^, our approach does not provide information about the source of undesirable animal welfare outcomes.

Furthermore, we believe that our model is applicable for approximating bleeding volumes with clear-cut configurations. Wounds cut by broad-head arrows used in archery hunting or knives (in forensic medicine), for instance, might mirror the configuration of cutting edges. Simple geometry involving known measurements can describe such bleeding volumes, allowing direct use of the theoretical model (3). However, the modeling can possibly be complicated, due to excessive pulmonary activity following injury, because strain at sharp wound edges in thoracic tissue can result in extended rupturing of the tissue.

Finally, our model does not predict instant incapacitation of large mammals, due to factors other than exsanguination, such as interference with the central nervous system or destruction of the spinal cord, which instantly paralyzes an animal. However, our model does support instant incapacitation when body mass is so small that tissue stress caused by the temporary cavity exceeds the elastic limit of vital internal organs, inflicting devastating and immediate wounding. Our interpretation of this phenomenon is that the volume of bleeding is so large relative to body mass that it leads to instantaneous shutdown of both pulmonary and circulatory systems.

### Uncertainties in the building of the model

In our approach, we assumed that the BTV constituted by the permanent wound cavity and the extravasation zone of the temporary wound cavity, conforms to a cylindrical shape of constant dimension, regardless of body mass, as long as the penetrated organ is large enough to avoid complete rupture, due to temporary cavitation. However, this assumption is an oversimplification. Fackler’s^35,37^ wound profile for expanding bullets, Schyma & Madea^59^, Gremse *et al*.^60^ and Trinogga *et al*.^58^ show that, although the permanent wound cavity basically is symmetrical around the centreline, it exhibits considerable longitudinal radial variation. Initially, before bullet expansion, the channel is narrow, but it increases rapidly as the leading edge of the bullet expands. As the expanded bullet decelerates and finally seizes up in the tissue, the BTV narrows and approximates the cross-sectional area of the bullet’s expanded area. Then, blood streams into the wound area from torn blood vessels (i.e. through a surface of a finite size). To simplify modelling, we assumed that the volume of extravascular blood can be approximated by simulating blood flow into an imaginary volume of tissue with a cylindrical shape with constant radius and surface area. It is extremely difficult to measure wound cavities in lungs in real time as severe puncturing of the pleura induces pneumothorax and lung collapse.

In our calculations, we use the BTV, i.e. the volume of the wound tract plus the extravasation zone, as equivalent to the volume of blood that is lost per time. However, the volume of the vessels in this tissue probably constitute a minor proportion of the total volume compared to other tissue types. Hence, we calibrated the model to data originating from ungulates felled by bullets penetrating both lungs. We presume that the well-perfused affected lung tissue, with its vessels torn apart by the direct and indirect damage of the penetrating bullet, will bleed diffusely, profusely and continuously into the alveoli and airways, the pleural cavity and the wound tract, and that the respiratory movements will facilitate removal of the blood, so that no efficient hemostasis occurs until the animal dies.

Our assumption of a constant dimension of the BTV irrespective of organ size is also a simplification of a complex course of events. The most important aspect of wounding is probably the permanent wound cavity^31,39,61^, where tissue laceration is irregularly distributed along the wound track and varies with drag, tissue type, expansion, yawing, fragmentation, or any supervening cause that affects the projectile before impact^28,37,62^. In spite of this irregularity, the permanent wound cavity is mainly the result of tissue crushing related to the presenting area of the projectile, rather than radial stretching related to the temporary cavity^31,37,39^. The temporary cavity can increase the extent of the permanent cavity, but elastic tissue like the lungs absorb much of the kinetic energy from the bullet and thereby lessen the damage, and thus the extent, of the permanent wound cavity^39,40,42^. Thus, for a given cross-sectional area of a bullet, we expect the radial size of the permanent wound cavity to be reasonably stable in lungs of various sizes. This applies as long as tissue mass is large enough to avoid complete rupture due to strain, caused by the temporary cavity, thus exceeding the elastic limit of the organ. This assumption is supported by the fact that Trinogga *et al*.^58^ did not discriminate among species when they used computed tomography to compare cross-sectional areas of wound cavities in lungs caused by different expanding bullets in 5 ungulate species ranging in size from roe deer to red deer.

Some variability in wound size could be expected due to the variety of calibers used in this study. Because >82% of the harvested animals were dispatched with calibers ranging between 6.5 and 8 mm, we did not expect any large effect of calibers in our modeling approach.

Our assumption that all animals travelled with the same speed is obviously wrong. We consider that the theoretical model (9) adequately describes time to incapacitation and therefore we believe that many species will be sufficiently embraced by the calibrated model (19) as exhibited by the comparison with real data. However, the precision of the model (19) will deteriorate for very “fast” or “slow” species. This deviation can simply be avoided by recalibrating the model (18) using average speed for the species in question.

Our theoretical model (9) was the main focus of this work. The empirical approach was just one way of solving the problem of the unknown radius. And, as such, this part of the modeling has clear limitations, even though we are comfortable with the calibrated output from the model. Therefore, we believe that field records of time to incapacitation could improve the precision of the model significantly.

## Conclusions

We consider our model to be a useful management tool for assessing whether an animal is wounded or not during a shooting operation. It enables monitoring animal welfare outcomes over time, for a given shooting program both within and among mammal species. By using our approach, management authorities can, at an early stage, determine whether a given shooting program has an acceptable wounding rate or not. In cases where improvement is needed, this can be addressed with targeted information or educational programs where appropriate. For hunters, a field guide based on the model could be used to allow hunters to assess whether a given animal has been wounded or not. We hope that our model will provide a basis for and inspire further research to inform managers and hunters on animal welfare outcomes and relative efficiency of firearms for shooting of wildlife species.

## Electronic supplementary material

Applied cartridges

Supplementary Dataset 1

## References

[1] Hampton, J. O., Forsyth, D. M., Mackenzie, D. I. & Stuart, I. G. A simple quantitative method for assessing animal welfare outcomes in terrestrial wildlife shooting: the European rabbit as a case study. *Anim. Welfare* **24**, 307–317. ISSN 0962-7286, 10.7120/09627286.24.3.307 (2015).

[2] Bradshaw EL, Bateson P (2000). Welfare implications of culling red deer (*Cervus elaphus*). Anim. Welfare.

[3] Knudsen SKA (2005). Review of the criteria used to assess insensibility and death in hunted whales compared to other species. Vet. J..

[4] Aebischer NJ, Wheatley CJ, Rose HR (2014). Factors Associated with Shooting Accuracy and Wounding Rate of Four Managed Wild Deer Species in the UK, Based on Anonymous Field Records from Deer Stalkers. PloS One.

[5] Dubois S (2017). International consensus principles for ethical wildlife control. Conserv. Biol..

[6] Mellor DJ, Littin KE (2004). Using science to support ethical decisions promoting humane livestock slaughter and vertebrate pest control. Anim. Welfare.

[7] Littin KE, Mellor DJ (2005). Strategic animal welfare issues: ethical and animal welfare issues arising from the killing of wildlife for disease control and environmental reasons. Rev. Sci. Tech. OIE..

[8] Newhook JC, Blackmore DK (1982). Electroencephalographic studies of stunning and slaughter of sheep and calves: part 1 - the onset of permanent insensibility in sheep during slaughter. Meat Sci..

[9] Vincent J-L, De Backer D (2013). Circulatory Shock. New Engl. J. Med..

[10] Gaieski, D. & Mikkelsen, M. Definition, classification, etiology, and pathophysiology of shock in adults in *UpToDate* Vol. 2017 (ed. Parsons, P. E.) UpToDate Inc. http://uptodate.com, Waltham, MA, USA (2018).

[11] Hollerman, J. J., Fackler, M. L., Coldwell, D. M. & Ben-Menachem, Y. Gunshot wounds: bullets, ballistics and mechanisms of injury. *Am. Roentgen Ray. Soc*., 155–685 (1990). 10.2214/ajr.155.4.2119095 2119095

[12] Newgard K (1992). The physiological effects of handgun bullets: the mechanisms of wounding and incapacitation. Wound Ballistics Rev..

[13] Maiden N (2009). Ballistics reviews: mechanisms of bullet wound trauma. Forensic Sci. Med. Pathol..

[14] Schmidt-Nielsen K (1984). Scaling. Why is Animal Size So Important?.

[15] Stokke, S., Arnemo, J. M., Söderberg, A. & Kraabøl, M. Wounding of carnivores – Understanding of concepts, status of knowledge and quantification. *NINA Report* **838** (2012).

[16] Gremse, C. & Rieger, S. Ergänzende Untersuchungen zur Tötungswirkung bleifreier Geschosse. Erweiterter Bericht zum Abschlussbericht vom 30.11.2012. Fachgebiet Wildbiologie, Wildtiermanagement & Jagdbetriebskunde (FWWJ). HNE Eberswalde Hochschule für nachhaltige Entwicklung (FH) (2014).

[17] Kanstrup, N., Balsby, T. J. S. & Thomas, V. G. Efficacy of non-lead rifle ammunition for hunting in Denmark. *Eur. J. Wildlife Res*. 10.1007/s10344-016-1006-0 (2016).

[18] Peters RH (1986). The Ecological Implications of Body Size.

[19] Niklas KJ (1994). Plant Allometry, The Scaling of Form and Process.

[20] Kleiber M (1932). Body size and metabolism. Hilgardia.

[21] Heusner, A. A. Size and power in mammals. *J. Exp. Biol*. **160** (1991). 10.1242/jeb.160.1.25 1960515

[22] Dodds PS, Rothman DH, Weitz JS (2001). Re-examination of the ‘3/4-law’ of metabolism. J. Theor. Biol..

[23] White CR, Seymour RS (2003). Mammalian basal metabolic rate is proportional to body mass2/3. Proc. Natl. Acad. Sci. USA.

[24] Glazier DS (2005). Beyond the ‘3/4-power law’: variation in the intra- and interspecific scaling of metabolic rates in animals. Biol. Rev. Camb. Philos. Soc..

[25] Savage VM (2004). The predominance of quarter power scaling in biology. Funct. Ecol..

[26] Takemoto K (2015). Heterogeneity of cells may explain allometric scaling of metabolic rate. Bio Systems.

[27] Lindstedt, S. L. & Schaeffer, P. J. Use of allometry in predicting anatomical and physiological parameters of mammals. *Lab. Anim*. **36** (2002). 10.1258/0023677021911731 11833526

[28] Kneubuehl, B. P., Coupland, R. M., Rothschild, M. A. & Thali, M. J. *Wound Ballistics, basics and applications* (Springer – Verlag Berlin Heidelberg, 2011).

[29] Callender GR, French RW (1935). Wound ballistics: studies in the mechanism of wound production by rifle bullets. Mil. Surg..

[30] Berlin R (1988). Terminal behaviour of deforming bullets. J. Trauma.

[31] Stefanopoulos PK, Filippakis K, Soupiou OT, Pazarakiotis VC (2014). Wound ballistics of firearm-related injuries-Part 1: Missile characteristics and mechanisms of soft tissue wounding. Int. J. Oral Max. Surg..

[32] Harvey EN, Whiteeley AH, Grundfest H, McMillen LHÆ (1946). Piezoelectric crystal measurements of pressure changes in the abdomen of deeply anaesthetized animals during the passage of HV missiles. Mil. Surgeon.

[33] Amato JJ, Rich NM (1972). Temporary cavity effects in blood vessel injury by high velocity missiles. J. Cardiovasc. Surg..

[34] Di Maio VJM (1999). Gunshot wounds, practical aspects of firearms, ballistics and forensic techniques.

[35] Fackler ML (2001). Wound profiles. Wound Ballistic Review.

[36] Felsmann MZ, Szarek J, Felsmann M, Babinska I (2012). Factors affecting temporary cavity generation during gunshot wound formation in animals – new aspects in the light of flow mechanics: a review. Vet. Med..

[37] Fackler ML (1988). Wound ballistics: a review of common misconceptions. AFTE J..

[38] Janzon, B., Hull, J. B. & Ryan, J. M. *Projectile, material interactions: soft tissue and bone* (eds Cooper, G. J., Dudley, H. A. F., Gann, D. S., Little, R. A. & Maynard, R. L.) 37–52 (Scientific foundations of trauma. Oxford: Butterworth Heinemann, 1997).

[39] MacPherson D (1994). Bullet Penetration - Modeling the Dynamics and Incapacitation Resulting from WoundTrauma.

[40] Karger, B. Forensic ballistics. *Forensic pathology reviews*, vol. 5 (ed. Tsokos, M.) 139–172 (Totowa, NJ: Humana Press;. p. 2008).

[41] Roberts GK (1998). The wounding effect of 5.56 mm/.223 law enforcement general purpose shoulder fired carbines compared with 12 GA. shotguns and pistol calibre weapons using 10% ordnance gelatine as a tissue simulant. Wound Ballistics Review.

[42] Caudell JN (2013). Review of wound ballistic research and its applicability to wildlife management. Wildlife Soc. B..

[43] Hejna P, Šafr M, Zátopková L (2012). The ability to act – Multiple suicidal gunshot wounds. J. Forensic Leg. Med..

[44] Evans, H. E. & de Lahunta, A. *Miller’s Anatomy of the Dog*. (Elsevier, Saunders 1979).

[45] Botha GSM (1957). The anatomy of phrenic nerve termination and the motor innervation of the diaphragm. Thorax.

[46] Röken, B. O. Jakt med kulvapen. Andra konsekvenser som kan uppstå, bl a jaktens effektivitet vad gäller djurskydd. Kolmården: Kolmårdens Djurpark (2006).

[47] Brown RP, Delp M, Lindstedt SL, Rhomberg LR, Beliles RP (1997). Physiological parameter values for physiologically based pharmacokinetic models. Toxicol. Ind. Health.

[48] Marx, H. *African bowhunting, the theory and the practise*. 4th Edition. South African Bowhunter’s Association Bowhunter Proficiency Certification (BProC) program (2013).

[49] Schmidt-Nielsen, K. Problems of scaling: locomotion and physiological correlates in Scale Effects in *Animal Locomotion* (ed. Pedley, T. J.) (New York, Academic Press 1977).

[50] Stahl WR (1967). Scaling of respiratory variables in mammals. J. Appl. Psychol..

[51] McGuill MW, Rowan AN (1989). Biological Effects of Blood Loss: Implications for Sampling Volumes and Techniques. ILAR Journal.

[52] Collier, B. R., Riordan, W. P., Nagy, R. J. & Morris, J. A. Wilderness trauma, surgical emergencies, and wound management in *Wilderness medicine*, *5th ed*. Philadelphia, PA, USA (ed. Auerbach, P. S) 475–504 (Mosby Elsevier 2007).

[53] Stokke, S., Brainerd, S. & Arnemo, J. M. Metal deposition of copper and lead bullets in moose harvested in Fennoscandia. *Wildlife Soc. B*. 10.1002/wsb.731 (2017).

[54] Hjorteviltregisteret. Fakta om artene. http://www.hjortevilt.no/fakta-om-artene/ (2016).

[55] Swenson JE, Sandegren F, Sὂderberg A, Franzén R (1995). Estimating the total weight of Scandinavian brown bears from field-dressed and slaughter weights. Wildlife Biol..

[56] Martin A (2017). Hunting of roe deer and wild boar in Germany: Is non-lead ammunition suitable for hunting?. PLoS ONE.

[57] McCann BE, Whitworth W, Newman RA (2016). Efficacy of non-lead ammunition for culling elk at Theodore Roosevelt National Park. Hum. Wildl. Interact..

[58] Trinogga A, Fritsch G, Hofer H, Krone O (2013). Are lead-free hunting rifle bullets as effective at killing wildlife as conventional lead bullets? A comparison based on wound size and morphology. Sci. Total Environ..

[59] Schyma C, Mades B (2012). Evaluation of the temporary cavity in ordnance gelatine. Forensic Sci. Int..

[60] Gremse F (2014). Performance of Lead-Free versus Lead-Based Hunting Ammunition in Ballistic Soap. PLoS ONE.

[61] Bartlett CS (2003). Clinical update: gunshot wound ballistics. Clin. Orthop. Relat. Res..

[62] Powers DB, Delo RI (2013). Characteristics of ballistic and blast injuries. Atlas of the Oral and Maxillofacial Surgery Clinics of North America.

