# Supplementary material for: Defining animal welfare standards in hunting: body mass determines thresholds for incapacitation time and flight distance
Source: Sci Rep. 2018 Sep 13;8:13786. doi: 10.1038/s41598-018-32102-0 (PMC6137050; doi:10.1038/s41598-018-32102-0)
Supplement: Supplementary file 1 — Applied cartridges [file 41598_2018_32102_MOESM1_ESM.pdf]

SUPPLEMENTARY INFORMATION

Defining animal welfare standards in hunting: body mass determines thresholds for incapacitation time and flight distance

Sigbjørn Stokke[1*], Jon M. Arnemo[2,3], Scott Brainerd[4,5], Arne Söderberg[6,7], Morten Kraabøl[1,8] & Bjørnar Ytrehus[1]

[1] Norwegian Institute for Nature Research, P.O. Box 5685 Torgard, NO-7485 Trondheim, Norway

[2] Inland Norway University of Applied Sciences, Campus Evenstad, Postboks 400, 2418 Elverum, Norway

[3] Department of Wildlife, Fish, and Environmental Studies, Swedish University of Agricultural Sciences, SE-90183 Umeå, Sweden

[4] Alaska Department of Fish and Game, Division of Wildlife Conservation, 1300 College Road, Fairbanks, AK 99701, USA

[5] Department of Ecology and Natural Resource Management, Norwegian University of Life Sciences, P.O. Box 5003, NO-1432 Ås, Norway

[6] National Veterinary Institute, SVA, SE-75189, Uppsala, Sweden

[7] County Administrative Board, Box 22067, 104 22 Stockholm, Sweden

[8] Multiconsult Norway AS, Postboks 265 Skøyen, NO-0213 Oslo, Norway

* Corresponding author: email: sigbjorn.stokke@nina.no tlf: 0047 90270350

APPLIED CARTRIDGES, ORDERED BY FREQUENCY OF USE

(NOMENCLATURE OF CARTRIDGES ACCORDING TO BARNES 2000).

Cartridge	Frequency
308 Winchester	0.3528
30-06 Springfield	0.2625
6,5x55 Swedish Mauser	0.1088
9.3x62mm Mauser	0.0859
7.62x53Rmm Russian	0.0272
338 Winchester Magnum	0.0225
8x57mmJS Mauser	0.0171
308 Norma Magnum	0.0167
45-70 Government	0.0144
375 H&H Magnum	0.0134
9,3x57mm Mauser	0.0115
300 Winchester Magnum	0.0091
7mm Remington Magnum	0.0080
270 Winchester	0.0064
9,3x74Rmm	0.0062
458 Winchester Magnum	0.0045
8x57mmJ Mauser	0.0037
9,3x64mm Brenneke	0.0035
404 Jeffery	0.0023
338 Lapua Magnum	0.0021
444 Marlin	0.0017
358 Norma Magnum	0.0016

358 Winchester	0.0014
300 Weatherby Magnum	0.0014
7x57Rmm Mauser	0.0014
6,5-284 Norma	0.0014
340 Weatherby Magnum	0.0012
8,2x53R Finnish	0.0012
7x64mm Brenneke	0.0012
7x57mm Mauser	0.0012
6,5x57mm Mauser	0.0012
35 Whelen	0.0010
416 Rigby	0.0008
375 Dakota	0.0006
416 Remington Magnum	0.0004
376 Steyr	0.0004
8x68mmS RWS	0.0004
300 H&H Magnum	0.0004
7mm-08 Remington	0.0004
460 Weatherby Magnum	0.0002
10,75x68mm Mauser	0.0002
416 Taylor	0.0002
378 Weatherby Magnum	0.0002
9,3x53Rmm Swiss	0.0002
338-06 JDJ	0.0002
338 Remington UltraMag	0.0002
7,65x53mm Mauser	0.0002

7mm-300 Weatherby Magnum	0.0002
270 Weatherby Magnum	0.0002
264 Winchester Magnum	0.0002
6.5-08 A-Square	0.0002
6,5-06	0.0002
